# Blebbistatin, a Myosin II Inhibitor, Exerts Antidepressant-Like Activity and Suppresses Detrusor Overactivity in an Animal Model of Depression Coexisting with Overactive Bladder

**DOI:** 10.1007/s12640-018-9948-5

**Published:** 2018-08-28

**Authors:** Andrzej Wróbel, Urszula Doboszewska, Ewa Rechberger, Małgorzata Bańczerowska-Górska, Piotr Czuczwar, Ewa Poleszak, Jarosław Dudka, Piotr Wlaź, Paweł Miotła, Edyta Wlaźlak, Tomasz Rechberger

**Affiliations:** 1 Second Department of Gynecology, Medical University of Lublin, Jaczewskiego 8, 20-090 Lublin, Poland; 2 Department of Animal Physiology, Institute of Biology and Biochemistry, Faculty of Biology and Biotechnology, Maria Curie-Skłodowska University, Akademicka 19, 20-033 Lublin, Poland; 3 Gynaecological and Obstetrics Hospital in Wałbrzych, Paderewskiego 10, 58-301 Wałbrzych, Poland; 4 Third Department of Gynecology, Medical University of Lublin, Jaczewskiego 8, 20-954 Lublin, Poland; 5 Chair and Department of Applied Pharmacy, Medical University of Lublin, Chodźki 1, 20-093 Lublin, Poland; 6 Chair and Department of Toxicology, Medical University of Lublin, Chodźki 8, 20-093 Lublin, Poland; 7 Clinic of Operative Gynecology and Gynecologic Oncology, 1st Department of Gynecology and Obstetrics, Medical University of Łódź, Wileńska 37, 94-029 Łódź, Poland

**Keywords:** Blebbistatin, 13-cis-Retinoic acid, Forced swim test, Overactive bladder, Rats

## Abstract

Overactive bladder (OAB) coexists with depression in women. Here, we assessed the effects of a 1-week treatment with blebbistatin, a myosin II inhibitor, on changes in behavior and detrusor overactivity (DO) symptoms induced by a 6-week administration of 13-cis-retinoic acid (13-cis-RA), with the aid of the forced swim test (FST), spontaneous locomotor activity test, and in vivo cystometric investigations in female Wistar rats. 13-cis-RA-induced depressive-like behavior and DO symptoms were associated with increased corticotropin-releasing factor (CRF) level in the plasma, prefrontal cortex (PFC), hippocampus (Hp), Barrington’s nucleus (BN), and urinary bladder. Moreover, 13-cis-RA decreased brain-derived neurotrophic factor (BDNF) and nerve growth factor (NGF) levels in plasma, PFC, Hp, and BN, while it increased BDNF and NGF levels in urinary bladder. Blebbistatin exerted antidepressant-like effect and attenuated changes in the cystometric parameters as well as the central and peripheral levels of CRF, BDNF, and NGF that were induced by 13-cis-RA, while it did not affect urine production, mean, systolic or diastolic blood pressure, or heart rate. The results point to blebbistatin as a potential treatment option for OAB coexisting with depression.

## Introduction

Overactive bladder (OAB) is a widely prevalent syndrome characterized by urinary urgency with or without urge incontinence, usually with frequency and nocturia (Haylen et al. 2012), which is more common in women than in men before the age of 60 years (Irwin et al. 2011). Interestingly, depression was found to be present in ca. 60% of women with the diagnosis of OAB (Melotti et al. 2018). Because there is no gold standard for the treatment of these coexisting conditions, therapeutic agents that target both OAB and depression are necessary.

Neurons of the Barrington’s nucleus (BN) constitute the pontine micturition center (Blok and Holstege 1998). They project to bladder motoneurons and innervate locus coeruleus (LC), which further projects to the prefrontal cortex (PFC) and hippocampus (Hp), brain regions which relevance to depression has been established (Palazidou 2012). Exposure to stress, a precipitant of depression (Gold et al. 2015), activates the LC through efferents from the corticotropin-releasing factor (CRF) system (Chandley and Ordway 2012). The high expression of CRF in the BN is believed to play a role in the regulation of bladder motility in response to stress (Valentino et al. 2011). In addition to CRF, neurotrophins (brain-derived neurotrophic factor (BDNF) and nerve growth factor (NGF)) may be involved in the pathophysiology underlying OAB coexisting with depression (Cruz 2014; Steers 2002). Neurotrophins may induce plastic changes of the neuronal circuits that govern bladder function (Ochodnicky et al. 2012). Elevated levels of NGF and BDNF were found in the bladder in animal models of OAB and in humans with OAB symptoms (Cruz 2014; Kashyap et al. 2018).

Myosins are a superfamily of actin-dependent motor proteins, which comprises 24 classes (numbered I to XXIV) (Foth et al. 2006). Class II myosins consist of skeletal, cardiac, smooth, and nonmuscle subclasses (Sellers 2000). Blebbistatin (BLEB) has been discovered with the aid of a high-throughput molecule screen for inhibitors of nonmuscle myosin II (NMMII) (Straight et al. 2003). Furthermore, it was demonstrated that BLEB inhibits both nonmuscle and muscle myosin (Newell-Litwa et al. 2015). An important fact is that BLEB was found to relax both rat and human bladder smooth muscle in vitro and significantly altered urodynamic parameters in vivo to values corresponding to decreased bladder overactivity (Zhang et al. 2011a). Moreover, BLEB was shown to relax bladder smooth muscles in a model of partial bladder outlet obstruction (PBOO) (Zhang et al. 2011b), which is associated with detrusor overactivity (DO).

In addition, myosins of class II play pre- and postsynaptic roles that are required for synapse function and facilitate synaptic plasticity (Kneussel and Wagner 2013). Myosins of this class regulate the rate of synaptic retrieval in a response to evoked release and determine the number of newly formed synaptic vesicles available for fusion (Chandrasekar et al. 2013). Given the emerging role of myosins in brain disorders (Newell-Litwa et al. 2015), here, we assessed the effects of BLEB treatment in a model reflecting OAB coexisting with depression.

## Materials and Methods

### Animals

All procedures were conducted in accordance with the European Communities Council Directive of 22 September 2010 (2010/63/EU) and Polish legislation acts concerning animal experimentations. The experimental procedures and protocols were approved by the First Local Ethics Committee at the Medical University of Lublin. The experiments were carried out on female Wistar rats, derived from the Center of Experimental Medicine at the Medical University of Lublin at the age of 4 weeks, initially weighing 200–225 g. Each rat was placed in a metabolic cage (3700M071, Tecniplast, West Chester, PA, USA) with free access to food and water. A natural light/dark cycle, temperature 22–24 °C and humidity 45–65% were maintained. A total of 90 female Wistar rats were used that were randomly divided into four groups; each group consisted of 15 animals that received:

1. Vehicle for 6 weeks and vehicle for 1 week (control group)
2. 13-cis-Retinoic acid (13-cis-RA) for 6 weeks and vehicle for 1 week (13-cis-RA group)
3. Vehicle for 6 weeks and BLEB for 1 week (BLEB group)
4. 13-cis-RA for 6 weeks and BLEB for 1 week (13-cis-RA + BLEB group)

### 13-cis-RA Administration

13-cis-RA (Tocris, Bristol, UK) was dissolved in darkness in a mixture of dimethyl sulfoxide (DMSO, Sigma-Aldrich, Saint Louis, MO, USA) and physiological saline (0.9% sodium chloride solution) at a ratio of 1:1, just before its administration. It was injected intraperitoneally (i.p.) at a dose of 1 mg/kg/day, at a volume of 1 ml/kg, on the alternating sides of the abdominal cavity, for a period of 6 weeks. The control group received a mixture of DMSO and physiological saline at 1:1 ratio i.p. The dose, route, and frequency of 13-cis-RA administration were based on previous studies in rodents showing depressive-like behavior after its administration (O'Reilly et al. 2006; Wróbel and Rechberger 2016). The levels of 13-cis-RA in plasma of rats belonging to 13-cis-RA and control groups are shown in Table 1. The level of 13-cis-RA in the 13-cis-RA group that was obtained in the present study corresponds to the level of 13-cis-RA in plasma of patients (0.37–1.09 μg/ml) that received 13-cis-RA p.o. starting at 0.5 mg/kg/day, escalating over 4 weeks to a maximum dose of 8 mg/kg/day (Kerr et al. 1982). It should be noted that administration of 13-cis-RA in humans is associated with depression (Bremner and McCaffery 2008).

**Table 1:** The effects of a 6-week administration of 13-cis-retinoic acid (13-cis-RA) (1 mg/kg/day, i.p.) and a 1-week administration of blebbistatin (BLEB) (125 nmol/kg/day, intra-arterial) on 13-cis-RA level in the plasma of rats

Treatment	Plasma level of 13-cis-RA (μg/ml)
Control	Not measurable
13-cis-RA	0.82 ± 0.04
BLEB	Not measurable
13-cis-RA + BLEB	0.72 ± 0.06

### Surgical Procedures

After 6 weeks of 13-cis-RA or vehicle administration, the surgical procedures were performed. All the surgical procedures were conducted under anesthesia with i.p. injection of 75 mg/kg of ketamine hydrochloride (Ketanest, Pfizer Pharma, Karlsruhe, Germany) and 15 mg/kg of xylazine (Sedazin, Biowet, Puławy, Poland). Rats were placed supine on a warming mattress (37 °C). The lack of spontaneous movement and withdrawal response to noxious toe pinch was viewed as an indication of an adequate depth of anesthesia. It was reported that ketamine in combination with xylazine does not abolish the micturition reflex in female rats (Cannon and Damaser 2001). The surgical procedures have been previously described in detail (Wróbel et al. 2015). In brief, the abdominal wall was opened through an approximately 10-mm vertical midline incision. A double lumen catheter was inserted through the apex of the bladder dome and fixed with 6-0 suture. The inner and outer diameters of the catheter were 0.28 and 0.61 mm, respectively. In the same session, the right femoral vein was catheterized through an inguinal approach. The catheters were tunneled subcutaneously (s.c.) and exteriorized in the retroscapular area, where they were connected with a plastic adapter, to avoid the risk of removal by the animal. The abdomen was closed in multiple layers. Anatomic layers were closed using 4/0 catgut sutures. The free ends of catheters were sealed with silk ligatures. The animals were injected s.c. with 100 mg/kg of cefazolin sodium hydrate (Biofazolin, Sandoz, Holzkirchen, Germany) to prevent urinary tract infection.

In the same session, in order to measure the blood pressure and for infusion of the test compound (BLEB) or vehicle into the bloodstream, the carotid artery was cannulated.

### Drugs

(±)-BLEB (Tocris) was dissolved in DMSO and administered as an intra-arterial bolus at a dose of 125 nmol/day for a period of 1 week. The control animals received volume-matched injections of vehicle. The dose of the drug was based on literature data in rodents which have been adjusted in our laboratory in preliminary experiments (Zhang et al. 2011a).

### The Measurement of Urine Production, Heart Rate, and Arterial Pressure

The animals were placed in metabolic cages (3700M071) for 24 h to measure urine production (UP), heart rate (HR), systolic blood pressure (SBP), mean blood pressure (MBP), and diastolic blood pressure (DBP).

### Conscious Cystometry

Cystometric investigations were performed in conscious unrestrained rats. The bladder catheter was connected via a three-way stopcock to a pressure transducer (FT03, Grass Technologies, West Warwick, RI, USA) and to a microinjection pump (CMA 100, CMA Microdialysis AB, Kista, Sweden). Conscious cystometry was performed by slowly filling the bladder with physiological saline at a constant rate 0.05 ml/min to elicit repetitive voiding. Micturition volumes were measured by means of a fluid collector attached to a force displacement transducer (FT03C). Both transducers were connected to a polygraph (7 DAG, Grass Technologies, West Warwick, RI, USA). Cystometry profiles and micturition volumes were recorded continuously on a Grass polygraph (Model 7E, Grass Technologies, West Warwick, RI, USA) and were determined graphically. The data were analyzed using a sampling rate of 10 samples/s. The measurements in each animal represent the average of five bladder micturition cycles after obtaining repetitive voiding. The following cystometric parameters were recorded: basal pressure (BP, cm H_2_O), threshold pressure (TP, cm H_2_O), micturition voiding pressure (MVP, cm H_2_O), voided volume (VV, ml), post-void residual (PVR, ml), volume threshold (VT, ml), voiding efficiency (VE, %), intercontraction interval (ICI, s), bladder contraction duration (BCD, s), relaxation time (RT, s), bladder compliance (BC, ml/cm H_2_O), detrusor overactivity index (DOI, cm H_2_O/ml), nonvoiding contractions amplitude (ANVC, cm H_2_O), nonvoiding contractions frequency (FNVC, times/filling phase), and volume threshold to elicit NVC (VTNVC, %).

### Locomotor Activity

The locomotor activity was assessed with the aid of a Digiscan apparatus: an Optical Animal Activity Monitoring System (Omnitech Electronics, Inc., Columbus, OH, USA). Activity chambers consisting of clear acrylic open field boxes were located in a room lit by a dim red light. The Digiscan system monitored animal locomotor activity via a grid of invisible infrared light beams. A number of equally spaced beams transversed the animal cage. The body of the animal placed in the Digiscan interrupted these beams revealing its position. The interruption of any beam was recorded as an activity score. Cumulative counts were compiled and downloaded every 15 min into the OMNIPRO data collection program. Prior to behavioral analysis, subjects were placed into activity chambers for a 15-min habituation period. Experiments were performed in a sound-proof room. Horizontal activity was assessed. This was defined as the total number of beam interruptions that occurred in the horizontal sensors during 1 h of measurement. All procedures were performed by a person blinded to the treatments.

### Forced Swim Test

The forced swim test (FST) was performed according to the method of Porsolt et al. (1977). Rats were placed individually into glass cylinders (height 65 cm, diameter 25 cm) containing 48 cm of water, maintained at 23–25 °C. Two swim sessions were carried out: an initial 15-min pretest was followed 24 h later by a 5-min test. Pretest was performed immediately after locomotor activity measurement, because locomotor activity test is non-invasive. Following both sessions, the rats were removed from the cylinders and returned to their home cages. Behavioral scoring was performed during the 5-min test session. The test sessions were videotaped from the side of the cylinders and scored using a behavioral sampling method by the person unaware of the treatment condition. The rat was judged to be immobile when it remained floating passively, performing slow motion movements to keep its head above the water.

### Tissue Processing

The blood was collected by cardiac puncture into tubes with EDTA-2Na (1 mg/ml) and was centrifuged (3000*g*) at 4 °C. The obtained plasma was stored at − 80 °C until biochemical analysis. The rats were killed by decapitation; their brains were rapidly dissected and immersed in cold (2–8 °C) saline. The PFC, Hp, and BN were dissected on a cold plate, immediately frozen on dry ice, and stored at − 80 °C until analysis. The urinary bladder was also dissected, immediately frozen on dry ice, and stored at − 80 °C until analysis.

### Biochemical Assessment

BDNF (Emax®ImmunoAssay System, Promega, USA), NGF (LifeSpan BioSciences, USA), and CRF (Mouse/Rat CRF-HS ELISA Kit, Alpco, Salem, NH, USA) levels were measured in plasma and tissue homogenates using enzyme-linked immunosorbent assay (ELISA), according to the manufacturer’s protocols. Each sample was measured in duplicate. The results are presented in picograms per milliliter for BDNF and NGF and in nanograms per milliliter for CRF.

### Determination of 13-Cis-RA Concentration

13-cis-RA concentration was assessed in plasma using reversed phase high-performance liquid chromatography (RP-HPLC). To prevent 13-cis-RA degradation, RP-HPLC steps were performed in darkness. The absorbance of the samples was identified at 354 nm, and 13-cis-RA concentration was determined by comparison to a standard curve.

### Statistics

The obtained data were evaluated by the two-way analysis of variance (ANOVA) followed by a Bonferroni post hoc test (where appropriate), using GraphPad Prism for Windows version 4 (GraphPad Software, San Diego, CA, USA). All results are presented as the mean ± SEM. *p* < 0.05 was considered as statistically significant with 95% confidence.

## Results

### The Effects of 13-cis-RA and BLEB Administration on Behavior in the Forced Swim Test (FST) and the Spontaneous Locomotor Activity of Rats

**Fig. 1:**
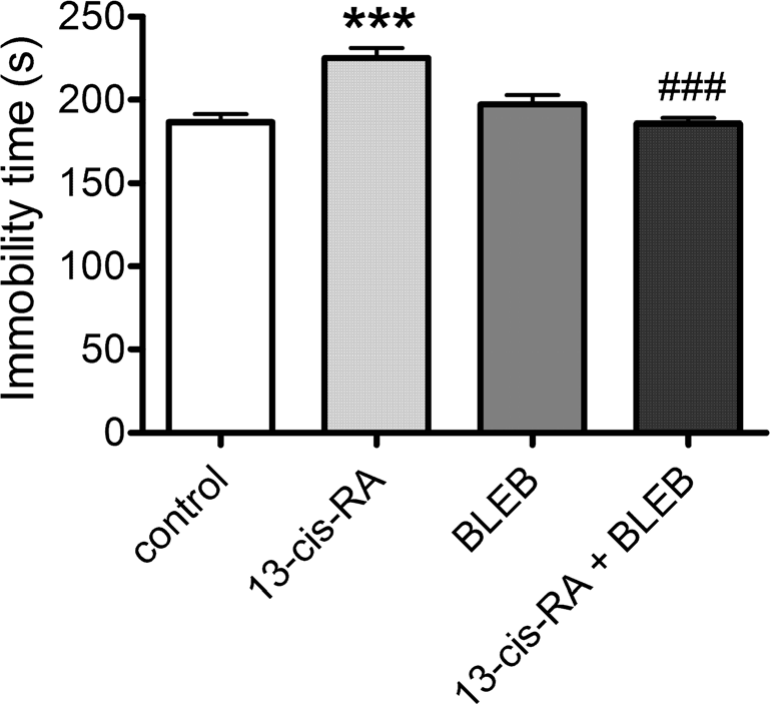
The effects of a 1-week administration of blebbistatin (BLEB) (125 nmol/day, intra-arterial) on changes in behavior induced by a 6-week administration of 13-cis-retinoic acid (13-cis-RA) in the forced swim test (FST). Data were analyzed by the two-way analysis of variance (two-way ANOVA) followed by a Bonferroni post hoc test; values are expressed as the mean ± SEM. ***p < 0.001 vs control; ###*p* < 0.001 vs 13-cis-RA. *N* = 15 in each group. 13-cis-RA effect [*F*(1,56 = 6.875, *p* = 0.0112], BLEB effect [*F*(1,56 = 7.830, *p* = 0.0112)], 13-cis-RA × BLEB interaction [*F*(1,56) = 23.93, *p* < 0.0001]

A 6-week administration of 13-cis-RA significantly increased the duration of immobility in the FST compared to rats that received vehicle for 6 weeks. A 1-week treatment with BLEB reversed this 13-cis-RA-induced effect and did not affect the immobility time in control rats (Fig. 1). There were no changes in the spontaneous locomotor activity between the examined groups (Table 2).

**Table 2:** The effects of a 6-week administration of 13-cis-RA (1 mg/kg/day, i.p.) and a 1-week administration of BLEB (125 nmol/day, intra-arterial) on the spontaneous locomotor activity of rats

Treatment	Spontaneous locomotor activity (cm)
Control	5452 ± 264.9
13-cis-RA	5658 ± 311.2
BLEB	5883 ± 317.8
13-cis-RA + BLEB	5954 ± 326.2

Data were analyzed by the two-way analysis of variance (two-way ANOVA), *n* = 15 in each group. 13-cis-RA effect [*F*(1,56) = 0.2050, *p* = 0.6525], BLEB effect [*F*(1,56) = 1.412, *p* = 0.2398), 13-cis-RA × BLEB interaction [*F*(1,56) = 0.04868, *p* = 0.8262)

### The Effects of 13-cis-RA and BLEB Administration on CRF Levels in Plasma, PFC, Hp, BN, and Urinary Bladder

**Fig. 2:**
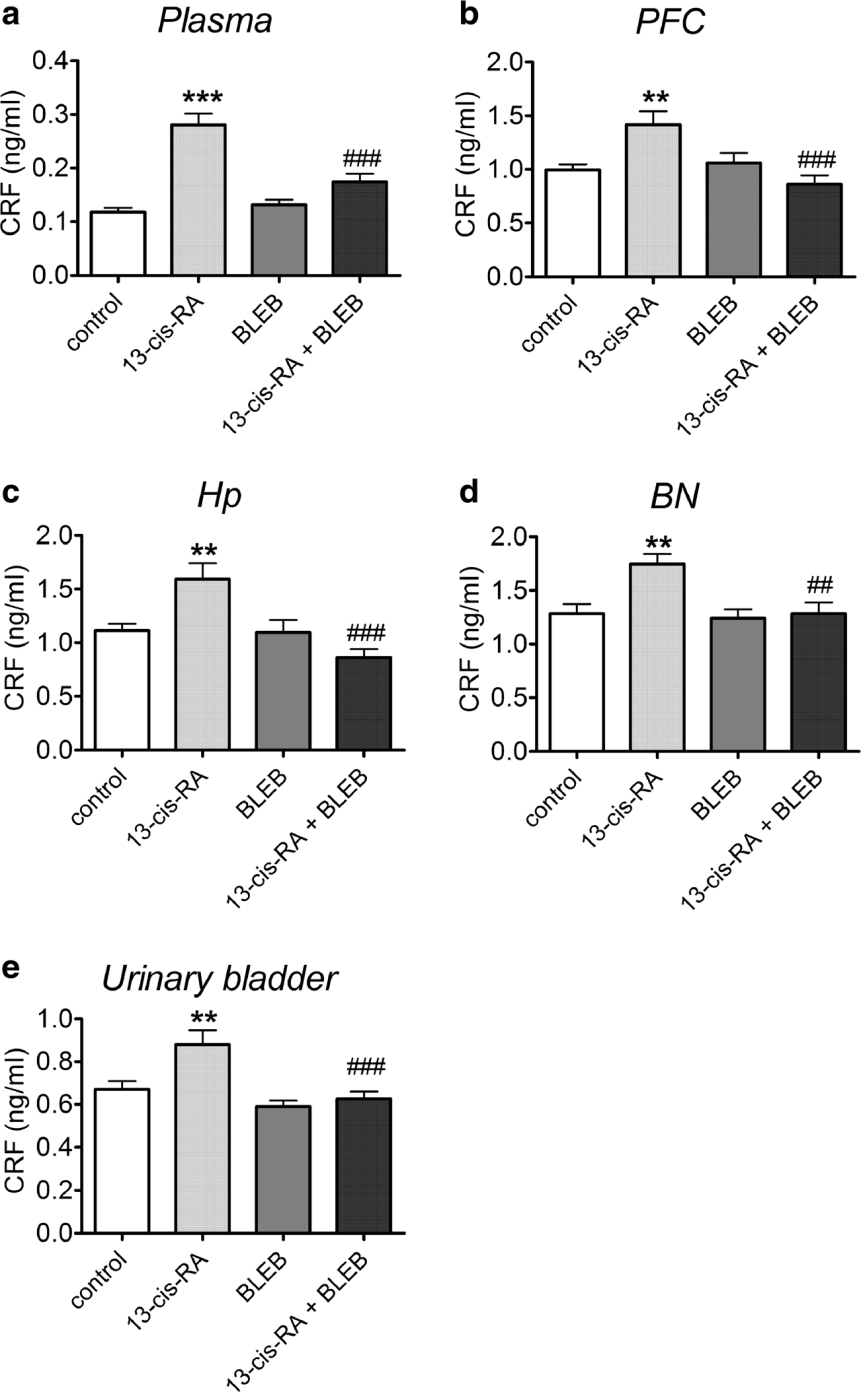
The effects of a 1-week administration of BLEB (125 nmol/day, intra-arterial) on changes in corticotropin releasing factor (CRF) concentration induced by a 6-week administration of 13-cis-RA in plasma (**a**), prefrontal cortex (PFC) (**b**), hippocampus (Hp) (**c**), Barrington nucleus’s (BN) (**d**), and urinary bladder (**e**). Data were analyzed by the two-way analysis of variance (two-way ANOVA) followed by a Bonferroni post hoc test; values are expressed as the mean ± SEM. **p < 0.01; ***p < 0.001 vs control; ##*p* < 0.01; ###*p* < 0.001 vs. 13-cis-RA. *N* = 15 in each group. CRF level in plasma: 13-cis-RA effect [*F*(1,56 = 48,08, *p* < 0.0001], BLEB effect [*F*(1,56 = 9.727, *p* = 0.0029)], 13-cis-RA × BLEB interaction [*F*(1,56) = 16.49, *p* = 0.0002]. CRF level in the PFC: 13-cis-RA effect [*F*(1,56 = 1.508, *p* = 0.2246], BLEB effect [*F*(1,56 = 7.269, *p* = 0.0092)], 13-cis-RA × BLEB interaction [*F*(1,56) = 11.61, *p* = 0.0012]. CRF level in the Hp: 13-cis-RA effect [*F*(1,56 = 1.280, *p* = 0.2628], BLEB effect [*F*(1,56 = 11.89, *p* = 0.0011)], 13-cis-RA × BLEB interaction [*F*(1,56) = 10.66, *p* = 0.0019]. CRF level in the BN: 13-cis-RA effect [*F*(1,56 = 7.489, *p* = 0.0083], BLEB effect [*F*(1,56 = 7.489, *p* = 0.0083)], 13-cis-RA × BLEB interaction [*F*(1,56) = 4.969, *p* = 0.0298]. CRF level in the urinary bladder: 13-cis-RA effect [*F*(1,56 = 7.450, *p* = 0.0085], BLEB effect [*F*(1,56 = 13.77, *p* = 0.0005)], 13-cis-RA × BLEB interaction [*F*(1,56) = 3.770, *p* = 0.0572]

13-cis-RA significantly increased CRF level in plasma (Fig. 2a), PFC (Fig. 2b), Hp (Fig. 2c), BN (Fig. 2d), and urinary bladder (Fig. 2e) of rats, while administration of BLEB significantly attenuated this 13-cis-RA-induced effect and did not affect the levels of CRF in rats that received vehicle for 6 weeks (Fig. 2a).

### The Effects of 13-cis-RA and BLEB Administration on BDNF Levels in Plasma, PFC, Hp, BN, and Urinary Bladder

**Fig. 3:**
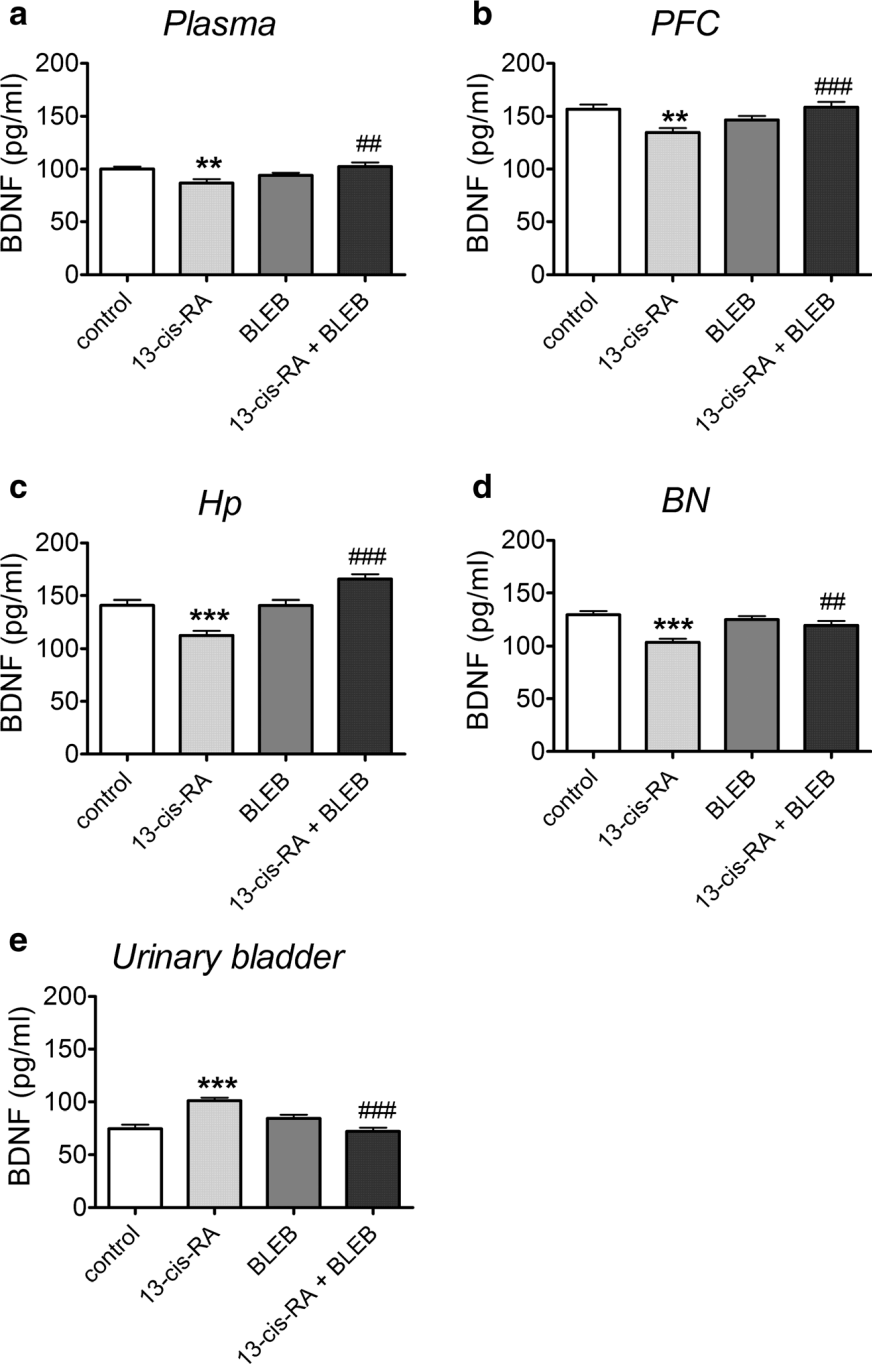
The effects of a 1-week administration of BLEB (125 nmol/day, intra-arterial) on changes in brain-derived neurotrophic factor (BDNF) concentration induced by a 6-week administration of 13-cis-RA in plasma (**a**), prefrontal cortex (PFC) (**b**), hippocampus (Hp) (**c**), Barrington nucleus’s (BN) (**d**), and urinary bladder (**e**). Data were analyzed by the two-way analysis of variance (two-way ANOVA) followed by a Bonferroni post hoc test; values are expressed as the mean ± SEM. **p < 0.01; ***p < 0.001 vs control; ##*p* < 0.01; ###*p* < 0.001 vs. 13-cis-RA. *N* = 15 in each group. BDNF level in plasma: 13-cis-RA effect [*F*(1,56 = 0.6131, *p* = 0.4369], BLEB effect [*F*(1,56 = 2.064, *p* = 0.1563)], 13-cis-RA × BLEB interaction [*F*(1,56) = 11.27, *p* = 0.0014]. BDNF level in the PFC: 13-cis-RA effect [*F*(1,56 = 1.283, *p* = 0.2621], BLEB effect [*F*(1,56 = 2.530, *p* = 0.1173)], 13-cis-RA × BLEB interaction [*F*(1,56) = 15.23, *p* = 0.0003]. BDNF level in the Hp: 13-cis-RA effect [*F*(1,56 = 0.1592, *p* = 0.6914], BLEB effect [*F*(1,56 = 30.97, *p* < 0.0001)], 13-cis-RA × BLEB interaction [*F*(1,56) = 31.67, *p* < 0.0001]. BDNF level in the BN: 13-cis-RA effect [*F*(1,56 = 18.97, *p* < 0.0001], BLEB effect [*F*(1,56 = 2.501, *p* = 0.1194)], 13-cis-RA × BLEB interaction [*F*(1,56) = 8.167, *p* = 0.0060]. BDNF level in the urinary bladder: 13-cis-RA effect [*F*(1,56 = 3.955, *p* = 0.0516], BLEB effect [*F*(1,56 = 7.359, *p* = 0.0089)], 13-cis-RA × BLEB interaction [*F*(1,56) = 30.88, *p* < 0.0001]

13-cis-RA significantly decreased BDNF level in plasma (Fig. 3a), PFC (Fig. 3b), Hp (Fig. 3c), and BN (Fig. 3d) of rats, while administration of BLEB significantly attenuated this 13-cis-RA-induced effect and did not affect the levels of BDNF in rats that received vehicle for 6 weeks. 13-cis-RA significantly increased BDNF level in the urinary bladder, while administration of BLEB significantly attenuated this 13-cis-RA-induced effect and did not affect the level of BDNF in control rats (Fig. 3e).

### The Effects of 13-cis-RA and BLEB Administration on NGF Levels in Plasma, PFC, Hp, BN, and Urinary Bladder

**Fig. 4:**
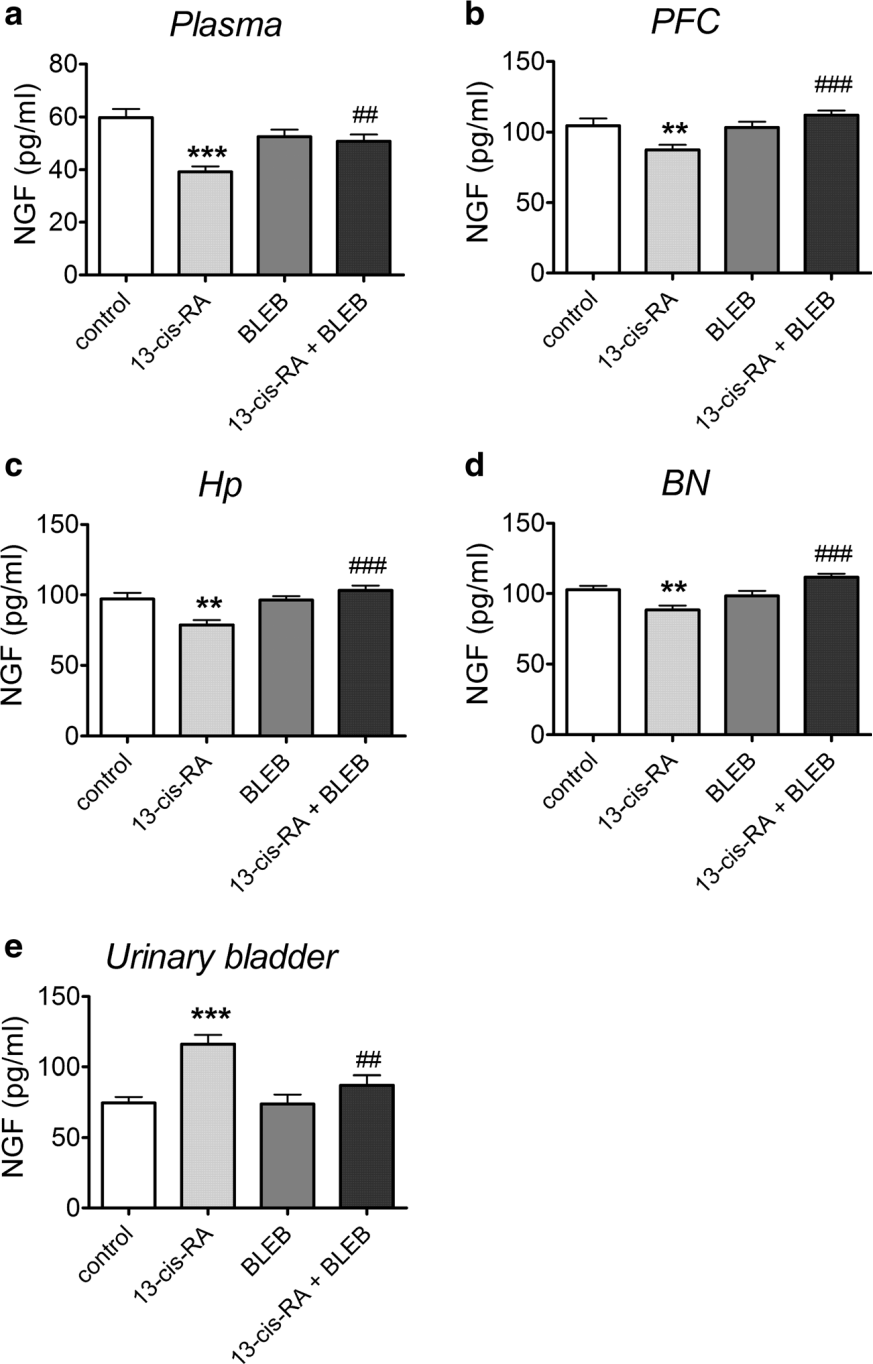
The effects of a 1-week administration of BLEB (125 nmol/day, intra-arterial) on changes in nerve growth factor (NGF) concentration induced by a 6-week administration of 13-cis-RA in plasma (**a**), prefrontal cortex (PFC) (**b**), hippocampus (Hp) (**c**), Barrington nucleus’s (BN) (**d**), and urinary bladder (**e**). Data were analyzed by the two-way analysis of variance (two-way ANOVA) followed by a Bonferroni post hoc test; values are expressed as the mean ± SEM. **p < 0.01; ***p < 0.001 vs control; ##*p* < 0.01; ###*p* < 0.001 vs. 13-cis-RA. *N* = 15 in each group. NGF level in plasma: 13-cis-RA effect [*F*(1,56 = 17.36, *p* = 0.0001], BLEB effect [*F*(1,56 = 0.7130, *p* = 0.4020)], 13-cis-RA × BLEB interaction [*F*(1,56) = 12.23, *p* = 0.0009]. NGF level in the PFC: 13-cis-RA effect [*F*(1,56 = 1.073, *p* = 0.3046], BLEB effect [*F*(1,56 = 8.030, *p* = 0.0064)], 13-cis-RA × BLEB interaction [*F*(1,56) = 9.766, *p* = 0.0028]. NGF level in the Hp: 13-cis-RA effect [*F*(1,56 = 2.644, *p* = 0.1096], BLEB effect [*F*(1,56 = 11,20, *p* = 0.0015), 13-cis-RA × BLEB interaction [*F*(1,56) = 12.89, *p* = 0.0007]. NGF level in the BN: 13-cis-RA effect [*F*(1,56 = 0.03155, *p* = 0.8596], BLEB effect [*F*(1,56 = 9.607, *p* = 0.0030)], 13-cis-RA × BLEB interaction [*F*(1,56) = 21.50, *p* < 0.0001]. NGF level in the urinary bladder: 13-cis-RA effect [*F*(1,56 = 19.59, *p* < 0.0001], BLEB effect [*F*(1,56 = 5.811, *p* = 0.0192)], 13-cis-RA × BLEB interaction [*F*(1,56) = 5.249, *p* = 0.0257]

13-cis-RA significantly decreased NGF level in plasma (Fig. 4a), PFC (Fig. 4b), Hp (Fig. 4c), and BN (Fig. 4d) of rats. Administration of BLEB significantly attenuated this 13-cis-RA-induced effect and did not affect the level of NGF in rats that received vehicle for 6 weeks. 13-cis-RA significantly increased NGF level in the urinary bladder of rats, while administration of BLEB significantly attenuated this 13-cis-RA-induced effect and did not affect the level of NGF in control rats (Fig. 4e).

### The Effects of 13-cis-RA and BLEB Administration on MBP, SBP, DBP, HR, and UP

Neither 13-cis-RA nor BLEB treatment significantly affected MBP, SBP, DBP, or HR in rats. There was a significant interaction between 13-cis-RA and BLEB with regard to UP (Table 3).

**Table 3:** The effects of a 6-week administration of 13-cis-RA (1 mg/kg/day, i.p.) and a 1-week administration of BLEB (125 nmol/day, intra-arterial) on mean blood pressure (MBP, mmHg), systolic blood pressure (SBP, mmHg), diastolic blood pressure (DBP, mmHg), heart rate (HR, beats/min), and urine production (UP, ml/day)

Treatment	MBP (mmHg)	SBP (mmHg)	DBP (mmHg)	HR (beats/min)	UP (ml/day)
Control	105.2 ± 3.22	111.2 ± 4.86	80.33 ± 2.25	253.4 ± 4.96	15.8 ± 0.69
13-cis-RA	114.0 ± 1.98	120.6 ± 3.75	87.60 ± 2.46	264.1 ± 4.10	17.5 ± 0.74
BLEB	109.3 ± 6.73	122.7 ± 4.81	85.80 ± 2.42	250.9 ± 5.60	17.4 ± 0.70
13-cis-RA + BLEB	108.9 ± 5.04	124.3 ± 3.91	84.00 ± 2.65	257.8 ± 4.65	15.6 ± 0.68

Data were analyzed by the two-way analysis of variance (two-way ANOVA), *n* = 15 in each group. MBP: 13-cis-RA effect [*F*(1,56) = 0.8283, *p* = 0.3667], BLEB effect [*F*(1,56) = 0.01174, *p* = 0.9141), 13-cis-RA × BLEB interaction [*F*(1,56) = 0.9936, *p* = 0.3231). SBP: 13-cis-RA effect [*F*(1,56) = 1.587, *p* = 0.2130], BLEB effect [*F*(1,56) = 3.030, *p* = 0.0872), 13-cis-RA × BLEB interaction [*F*(1,56) = 0.7979, *p* = 0.3755). DBP: 13-cis-RA effect [*F*(1,56) = 1.240, *p* = 0.2702], BLEB effect [*F*(1,56) = 0.1449, *p* = 0.7049), 13-cis-RA × BLEB interaction [*F*(1,56) = 3.410, *p* = 0.0701). HR: 13-cis-RA effect [*F*(1,56) = 3.276, *p* = 0.0756], BLEB effect [*F*(1,56) = 0.8191, *p* = 0.3693), 13-cis-RA × BLEB interaction [*F*(1,56) = 0.1527, *p* = 0.6974). UP: 13-cis-RA effect [*F*(1,56) = 6.243, *p* = 0.9607], BLEB effect [*F*(1,56) = 6.243, *p* = 0.8935), 13-cis-RA × BLEB interaction [*F*(1,56) = 6.243, *p* = 0.0154)

### The Effects of 13-cis-RA and BLEB Administration on Cystometric Parameters

**Fig. 5:**
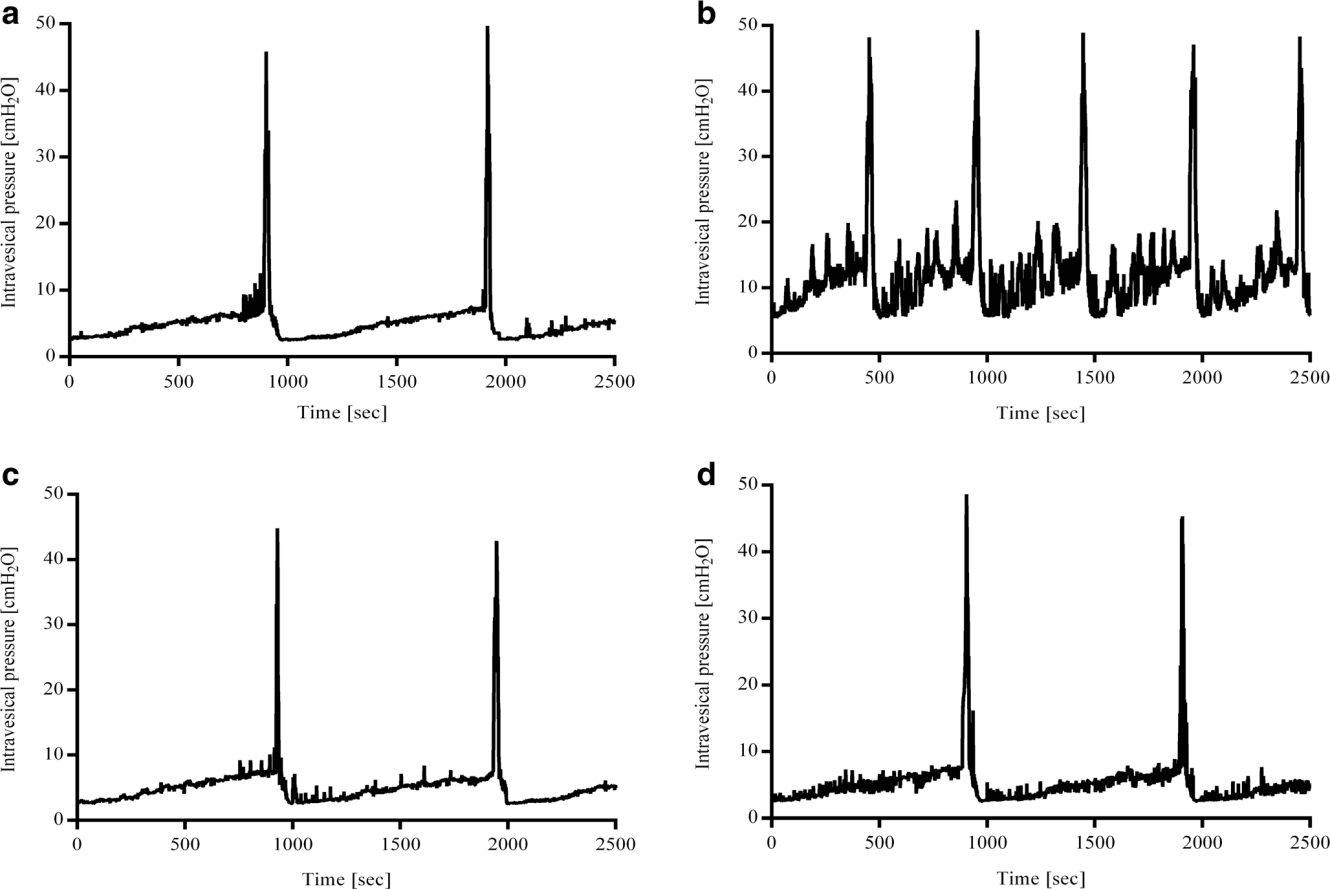
Representative cystometrograms in control (**a**), 13-cis-RA (**b**), BLEB (**c**), and 13-cis-RA + BLEB (**d**) rats

13-cis-RA significantly increased BP, DOI and FNVC parameters. Administration of BLEB significantly attenuated this 13-cis-RA-induced effect and did not affect these parameters in control rats. Moreover, 13-cis-RA significantly decreased VV and ICI parameters. Administration of BLEB significantly attenuated this 13-cis-RA-induced effect and did not affect these parameters in control rats. Furthermore, 13-cis-RA significantly decreased VTNVC. Administration of BLEB decreased VTNVC in control rats, but it increased VTNVC in 13-cis-RA-treated rats. In addition, BLEB administration decreased BC in control rats, while it increased BC in 13-cis-RA-treated rats (Table 4). Representative cystometrograms are shown in Fig. 5.

**Table 4:** The effects of a 6-week administration of 13-cis-RA (1 mg/kg/day, i.p.) and a 1-week administration of BLEB (125 nmol/day, intra-arterial) on basal pressure (BP, cm H_2_O), voided volume (VV, ml), post-void residual (PVR, ml), intercontraction interval (ICI, s), bladder compliance (BC, ml/cm H_2_O), detrusor overactivity index (DOI, cm H_2_O/ml), nonvoiding contractions frequency (FNVC, times/filling phase), and volume threshold to elicit NVC (VTNVC, %)

Treatment	BP, cm H_2_O	VV, ml	PVR, ml	ICI, s	BC, ml/cm H_2_O	DOI, cm H_2_O/ml	FNVC, times/filling phase	VTNVC, %
Control	2.37 ± 0.13	0.82 ± 0.03	0.05 ± 0.004	943.8 ± 26.52	0.21 ± 0.006	66.60 ± 6.75	0.41 ± 0.05	85.47 ± 4.72
13-cis-RA	3.48 ± 0.26**	0.61 ± 0.04**	0.06 ± 0.004	813.9 ± 28.65*	0.18 ± 0.006	143.0 ± 13.79***	2.34 ± 0.28***	58.12 ± 5.00***
BLEB	2.53 ± 0.24	0.71 ± 0.06	0.06 ± 0.004	891.3 ± 43.14	0.17 ± 0.008*	64.07 ± 5.81	0.99 ± 0.22	63.35 ± 4.32**
13-cis-RA + BLEB	2.58 ± 0.23#	0.80 ± 0.05#	0.07 ± 0.006	1022.0 ± 52.33###	0.22 ± 0.012##	74.07 ± 5.99###	1.39 ± 0.20##	93.26 ± 5.67###

*p < 0.05, **p < 0.01, ***p < 0.001 vs control, #*p* < 0.05, ##*p* < 0.01, ###*p* < 0.001 vs. 13-cis-RA, by the two-way analysis of variance (two-way ANOVA) followed by a Bonferroni post hoc test, *n* = 15 in each group. BP: 13-cis-RA effect [*F*(1,56) = 6.413, *p* = 0.0142], BLEB effect [*F*(1,56) = 2.655, *p* = 0.1088), 13-cis-RA × BLEB interaction [*F*(1,56) = 5.417, *p* = 0.0236)]. VV: 13-cis-RA effect [*F*(1,56) = 1.410, *p* = 0.2401], BLEB effect [*F*(1,56) = 2.655, *p* = 0.3603), 13-cis-RA × BLEB interaction [*F*(1,56) = 0.8508, *p* = 0.0042)]. PVR: 13-cis-RA effect [*F*(1,56) = 1.759, *p* = 0.1902], BLEB effect [*F*(1,56) = 5.545, *p* = 0.0221), 13-cis-RA × BLEB interaction [*F*(1,56) = 0.5690, *p* = 0.4538)]. ICI: 13-cis-RA effect [*F*(1,56) = 1.759, *p* = 0.1902], BLEB effect [*F*(1,56) = 5.545, *p* = 0.0221), 13-cis-RA × BLEB interaction [*F*(1,56) = 0.5690, *p* = 0.4538)]. BC: 13-cis-RA effect [*F*(1,56) = 1.657, *p* = 0.2033], BLEB effect [*F*(1,56) = 0.09135, *p* = 0.7636), 13-cis-RA × BLEB interaction [*F*(1,56) = 17.62, *p* < 0.0001)]. DOI: 13-cis-RA effect [*F*(1,56) = 24.43, *p* < 0.0001], BLEB effect [*F*(1,56) = 16.71, *p* = 0.0001), 13-cis-RA × BLEB interaction [*F*(1,56) = 0.5690, *p* = 0.0004)]. FNVC: 13-cis-RA effect [*F*(1,56) = 30.00, *p* < 0.0001], BLEB effect [*F*(1,56) = 0.7746, *p* = 0.3826), 13-cis-RA × BLEB interaction [*F*(1,56) = 12.96, *p* = 0.0007)]. VTNVC: 13-cis-RA effect [*F*(1,56) = 0.06678, *p* = 0.7970], BLEB effect [*F*(1,56) = 1.727, *p* = 0.1941), 13-cis-RA × BLEB interaction [*F*(1,56) = 33.41, *p* < 0.0001)]

## Discussion

We have previously shown that treatment with 13-cis-RA induces depressive-like behavior in female rats as measured by the increased immobility time in the FST as well as changes in cystometric parameters analogous to DO symptoms (Wróbel and Rechberger 2016). Furthermore, we have demonstrated that antimuscarinic drugs—representatives of first- (solifenacin) and second (mirabegron)-line treatments for OAB as well as a representative of proposed novel treatments for OAB—a reversible CRF_1_ antagonist, SN003, attenuated DO symptoms induced by 13-cis-RA (Wróbel and Rechberger 2016). Moreover, depressive-like behavior induced by 13-cis-RA was attenuated by a tricyclic antidepressant, imipramine, and by SN003 (Wróbel et al. 2017a). Thus, our previous findings point to 13-cis-RA administration as an animal model reflecting OAB coexisting with depression.

Using the proposed 13-cis-RA model, here, we show for the first time the antidepressant-like effect following administration of BLEB, a myosin II inhibitor. With the aid of partial bladder outlet obstruction model, BLEB has been suggested as a novel strategy to regulate detrusor contractily (Zhang et al. 2011b). Here, antidepressant-like effect of BLEB was parallel to attenuation of DO symptoms, which further highlights that the compound may represent a new treatment option for DO. Because BLEB inhibits both nonmuscle and muscle myosins (Newell-Litwa et al. 2015), and thus can affect cardiac muscle contractions, we assessed cardiovascular parameters. We found no changes in heart rate, mean, systolic, or diastolic blood pressure after administration of 13-cis-RA or BLEB. Taken together, the obtained results point to BLEB as a potential treatment strategy for OAB coexisting with depression, possibly with favorable cardiovascular profile.

Moreover, treatment with BLEB normalized changes in factors which are recognized as pathophysiological components of both depressive (Nestler et al. 2002) and voiding disorders (Klausner and Steers 2004; Wróbel et al. 2017b), i.e., CRF, BDNF and NGF, in central and peripheral compartments. PFC and Hp are among well-studied regions of the brain that exhibit retinoic acid signaling with regard to depressive disorders (Bremner and McCaffery 2008). In the present study, 13-cis-RA-induced depressive-like behavior and DO symptoms were associated with increased CRF level in plasma, PFC, Hp, BN, and urinary bladder, while administration of BLEB attenuated these changes. We have previously observed that co-occurrence of depressive and DO symptoms induced by 13-cis-RA was parallel to higher concentration of CRF in hypothalamus, amygdala, and plasma (Wróbel et al. 2017a). A compelling evidence points to disturbances within CRF system in animal models of depression and in depressed patients (Nestler et al. 2002). Data indicate increased production of CRF in brain regions such as the PFC and Hp in depression, while antidepressant action has been linked to normalization of raised CRF level (Aubry 2013; Holsboer 2000). Data on CRF involvement in voiding disorders are more conflicting. Both excitatory and inhibitory effects of CRF on micturition have been shown (Valentino et al. 2011). An inhibitory influence of CRF on micturition was suggested based on findings that chemical activation of BN induced bladder contractions that were increased after intrathecal administration of a CRF antagonist (Lang and Borgwardt 2013), while intrathecally administered CRF decreased BN-stimulated bladder contractions (Pavcovich and Valentino 1995). Moreover, social stress, which causes urinary retention, up-regulated CRF protein and mRNA levels in BN (Wood et al. 2009). We have found that attenuation of DO symptoms following administration of a reversible CRF_1_ antagonist, SN003, was associated with reduction of raised by 13-cis-RA administration levels of CRF in plasma, amygdala, and hypothalamus (Wróbel et al. 2017a). Thus, changes of CRF system may represent a common pathophysiological underpinning of depression and OAB as well as their therapeutic mechanisms.

BDNF and NGF are among the best characterized neurotrophins in terms of its role in synaptic plasticity, as well as its potential role in the pathology and treatment of a variety of psychiatric disorders including depression (Autry and Monteggia 2012; Lang and Borgwardt 2013). BDNF was found to be decreased in plasma of depressed patients (Polyakova et al. 2015). Moreover, post-mortem studies of suicide subjects have demonstrated decreased BDNF expression at mRNA and protein level in the PFC and Hp (Dwivedi et al. 2003). Furthermore, post-mortem studies showed increased BDNF levels in the PFC and Hp after long-term antidepressant use (Autry and Monteggia 2012). On the other hand, NGF level was found to be elevated in bladders in pathological states such as OAB, bladder pain syndrome, idiopathic and neurogenic DO, bladder oversensitivity, and bladder outflow obstruction. Moreover, successful treatment with antimuscarinics was associated with reduction of raised urinary NGF levels (Seth et al. 2013). In animal models of partial urethral obstruction, chemical cystitis, and spinal cord injury, pretreatment with antibodies against NGF or its receptor prevented urinary frequency and unstable contractions. In contrast, intravesical NGF administration caused unstable detrusor contractions (Steers 2002). Thus, in addition to alteration of CRF system, changes of neurotrophic mechanisms may be a common pathophysiological trait linking OAB to depression.

## Conclusions

We report for the first time the antidepressant-like effect following the myosin II inhibitor, BLEB, administration, which is parallel to attenuation of changes in the cystometric parameters as well as central and peripheral levels of CRF, BDNF, and NGF, that were altered by 13-cis-RA. The results suggest BLEB as a potential treatment option for OAB coexisting with depression. The study further highlights that DO and depressive-like behavior may share common pathophysiology, which raises the possibility that agents targeting factors altered by both conditions, such as BLEB, may be beneficial in case of coexisting OAB and depression.
